# New Cerebral Microbleeds and Mechanism of Post-Thrombolysis Remote Intracerebral Hemorrhage: “Red Meets White” Revisited^†^

**DOI:** 10.3389/fneur.2015.00203

**Published:** 2015-09-15

**Authors:** Ashkan Shoamanesh, Shenqiang Yan, Andreas Charidimou

**Affiliations:** ^1^Population Health Research Institute, McMaster University, Hamilton, ON, Canada; ^2^Department of Neurology, School of Medicine, The 2nd Affiliated Hospital of Zhejiang University, Hangzhou, China; ^3^UCL Institute of Neurology, London, UK; ^4^Hemorrhagic Stroke Research Program, Department of Neurology, Massachusetts General Hospital Stroke Research Center, Harvard Medical School, Boston, MA, USA

**Keywords:** acute stroke, cerebral microbleeds, cerebral small vessel disease, cerebral amyloid angiopathy, intracerebral hemorrhage, thrombolysis

Intravenous thrombolytic therapy in acute ischemic stroke patients is complicated by intracerebral hemorrhage (ICH) at a site remote from the infarcted area in roughly 2–3% of cases (1, 2). Historically, the etiology underlying these was proposed to be hemorrhagic infarction at a distant unrecognized silent focus of ischemia from multiple emboli. However, the use of diffusion-weighted imaging has demonstrated clear examples of remote intracerebral hemorrhage (rICH) occurring at sites devoid of ischemia, signifying alternate contributory mechanisms (3). Cerebral microbleeds (CMBs) are markers of bleeding-prone microangiopathies – most commonly hypertensive arteriopathy and cerebral amyloid angiopathy (CAA) (4) – that are visualized on T2*-weighted magnetic resonance imaging (MRI). Pathological studies have demonstrated intact erythrocytes underlying 13% of CMBs (5) implying that a subset of these lesions reflect acute or subacute areas of microhemorrhage. Fittingly, radiographic studies have demonstrated development of new CMBs in 5–13% of acute ischemic stroke patients within the first week after symptom onset (6–8). It is hence biologically plausible that thrombolysis-induced expansion of rapidly appearing CMBs might be the cause underlying a proportion of rICH in acute ischemic stroke patients. In this Opinion piece, we explored this hypothesis by pooling available evidence from relevant MRI patient cohorts with acute ischemic stroke.

Two recent studies from east-Asian centers including a total of 345 patients have assessed the risk of rICH in patients who develop new post-stroke CMBs. Both studies used exclusively intravenous thrombolysis with rtPA: the dose used was 0.6 mg/kg in one study (7) and 0.9 mg/kg in the other (8). Overall, 129 (39%) of the patients had CMBs on pre-treatment baseline MRI and 17 (5%) developed new CMBs at 24 h post-thrombolysis. Post-thrombolysis rICH occurred in 2% (*n* = 7) of the entire population. In fixed effects pooled meta-analysis of the data, patients who developed new CMBs had a significantly increased risk of rICH than patients without new CMBs (odds ratio (OR) 16.15, 95% CI 3.72–70.18, *p* < 0.0001; Figure 1). The results were consistent in sensitivity analysis using a random effects model.

**Figure 1:**
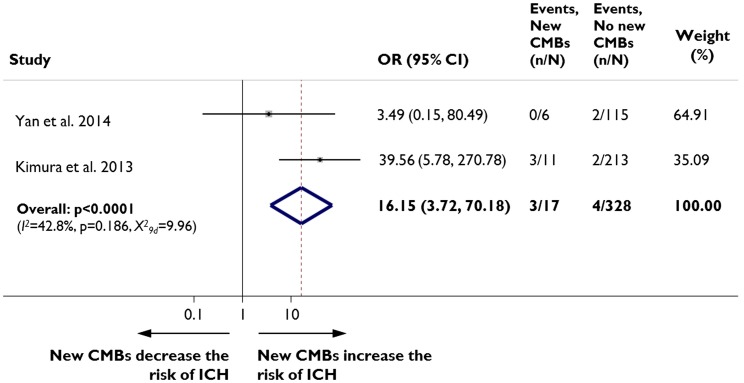
**New cerebral microbleeds and remote (i.e., extra-ischemic) intracerebral hemorrhage**. Meta-analysis of the association between symptomatic remote intracerebral hemorrhage (ICH) risk in patients with acute ischemic stroke treated with intravenous thrombolysis, in relation to the presence of new cerebral microbleeds (CMBs) on MRI. Moderate heterogeneity was detected across the different studies pooled in the meta-analysis. Two authors search PubMed and extracted relevant data for the analysis. We quantified the strength of the association between new CMBs and ICH using odds ratios (OR) and their corresponding 95% CIs, with the inverse variance method for weighting. We assessed statistical heterogeneity using I-squared statistics and also visually through inspection of the forest plot. We repeated all analyses using random effects models. Meta-analyses were performed using Stata 11.2 (StataCorp LP, Texas).

These findings, although preliminary, suggest that thrombolysis-induced expansion of new CMBs might account for a proportion of rICH in acute ischemic stroke patients. Post-thrombolysis rICH has been previously documented to occur at a site of CMB (9) and meta-analyses have suggested elevated risk of any post-thrombolysis symptomatic intracerebral hemorrhage in patients with CMBs (10, 11). However, whether these lesions – detected on baseline pre-thrombolysis MRI – were acute CMBs or simply a chronic marker of underlying hemorrhage-prone microangiopathies in the brain is uncertain, as thus far only chronic-subacute CMBs have been proposed to possibly have a distinctive MRI signature (12).

Two cohorts published in the last year have attempted to characterize clinical predictors of rICH (1, 2). In the Safe Implementation of Treatments in Stroke-International Stroke Register (SITS-ISTR) prior stroke and older age were independently associated with rICH. However, the lack of robust associations with traditional ischemic risk factors led the authors to postulate whether another undetected mechanism, including CAA (13), was at play (2). Conversely, prior transient ischemic attack (TIA) was the only clinical predictor of rICH in an Australian cohort (1). Although, this observation could support the notion that rICH occurs from hemorrhagic transformation of unrecognized acute or subacute ischemic infarcts, patients with CAA often experience transient focal neurological episodes that can mimic TIA, and are highly predictive of future lobar ICH (14).

Together, these observations raise the possibility that multiple etiologies (both primary hemorrhagic and primary ischemic) likely contribute to the pathogenesis of post-thrombolysis rICH. They also demonstrate the rapidly evolving nature of microbleeds in the acute phase of ischemic stroke, suggesting a potential role of an active small vessel microangiopathic process (15). As our pooled analysis is unadjusted, it remains to be determined whether the association between new CMBs and rICH is indeed an independent one or rather simply an indirect association due to common underlying pathophysiology, such as small vessel disease, stroke-induced acute hypertensive response, or neurovascular unit dysfunction from up regulation of inflammatory cascades. Future larger studies that incorporate a comprehensive assessment of both clinical and MRI predictors of rICH, as well as circulating markers of inflammation, would further elucidate this hypothesis.

## Author Contributions

Study Concept: AS and AC. Acquisition of data: AS, SY, and AC. Statistical Analysis: AC. Drafting of the manuscript: AS; Revising the manuscript for content: AC and SY.

## Conflict of Interest Statement

The authors declare that the research was conducted in the absence of any commercial or financial relationships that could be construed as a potential conflict of interest.
